# Supplementary material for: Semi-Quantitative [18F]FDG-PET/CT ROC-Analysis-Based Cut-Offs for Aortitis Definition in Giant Cell Arteritis
Source: Int J Mol Sci. 2022 Dec 8;23(24):15528. doi: 10.3390/ijms232415528 (PMC9779846; doi:10.3390/ijms232415528)
Supplement: Supplementary file 1 [file ijms-23-15528-s001.zip › ijms-1992432-supplementary.pdf]

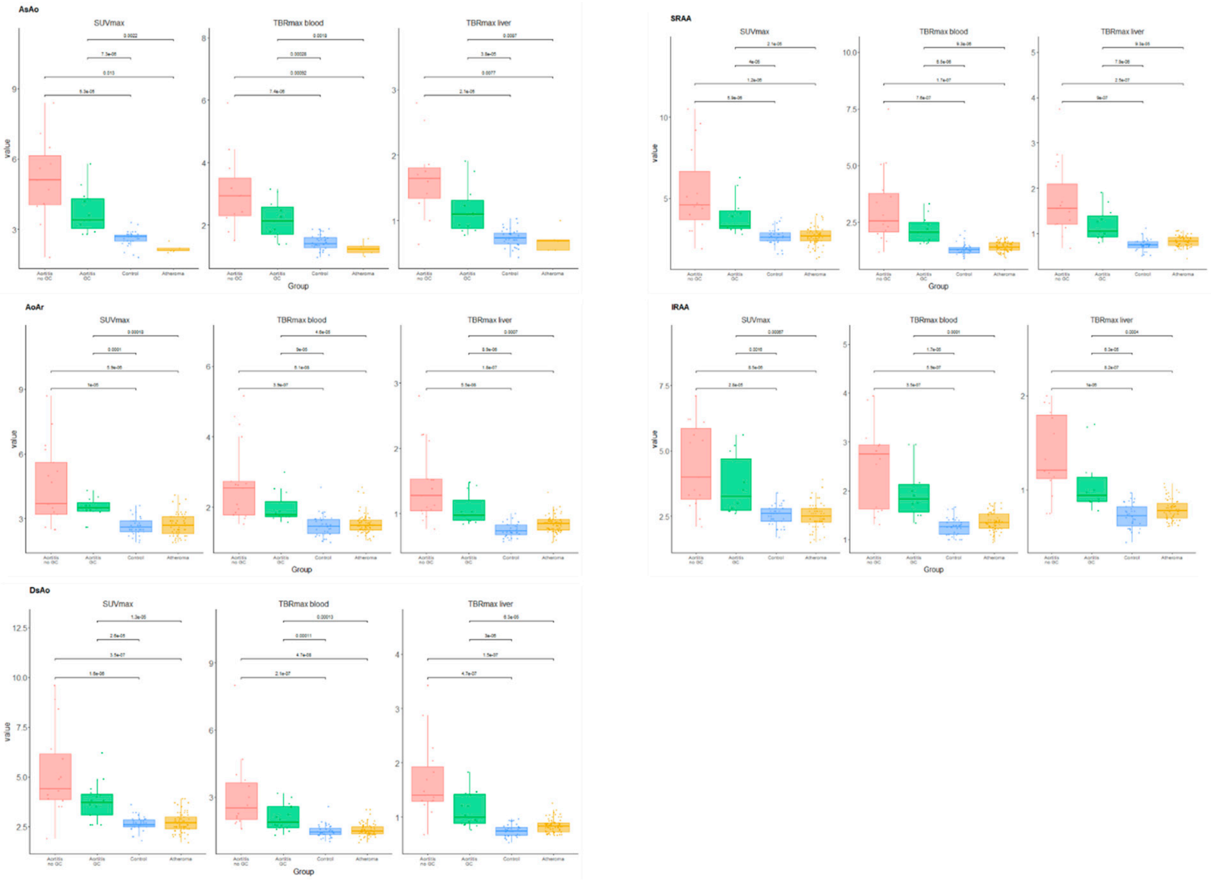

Supplemental Figure S1: SUV_{max}, TBR_{blood} and TBR_{liver} values of aortic signal in each aortic segment in aortitis without and with corticosteroid, aortic atheroma and control groups in the ascending thoracic aorta (AsAo), aortic arch (AoAr), descending thoracic aorta (DsAo), suprarenal abdominal aorta (SRAA) and infrarenal abdominal aorta (IRAA).

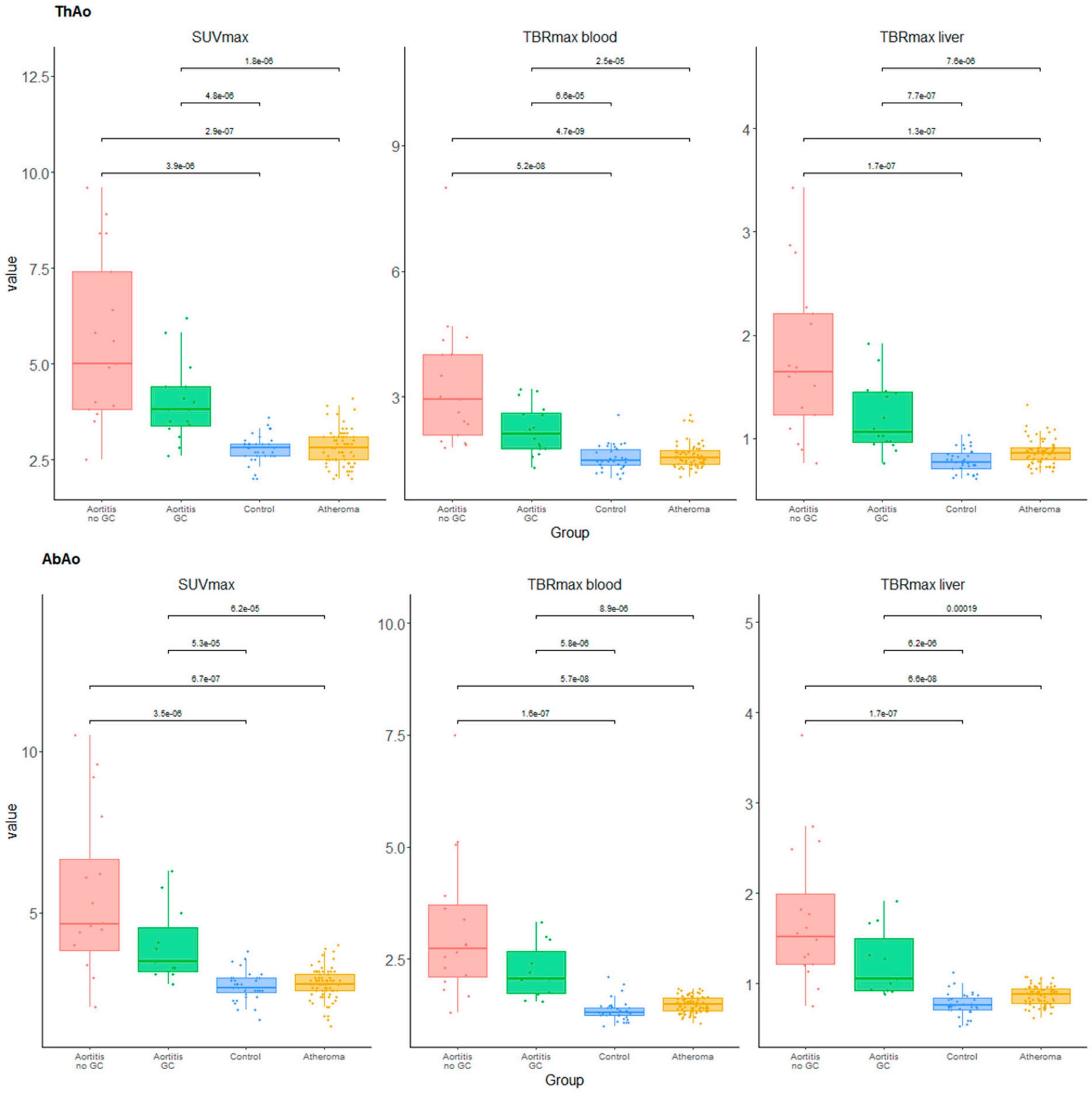

Supplemental Figure S2: Distribution of SUV_{max}, TBR_{blood} and TBR_{liver} values of aortic signal in thoracic (ThAo) and abdominal (AbAo) aortic segments.

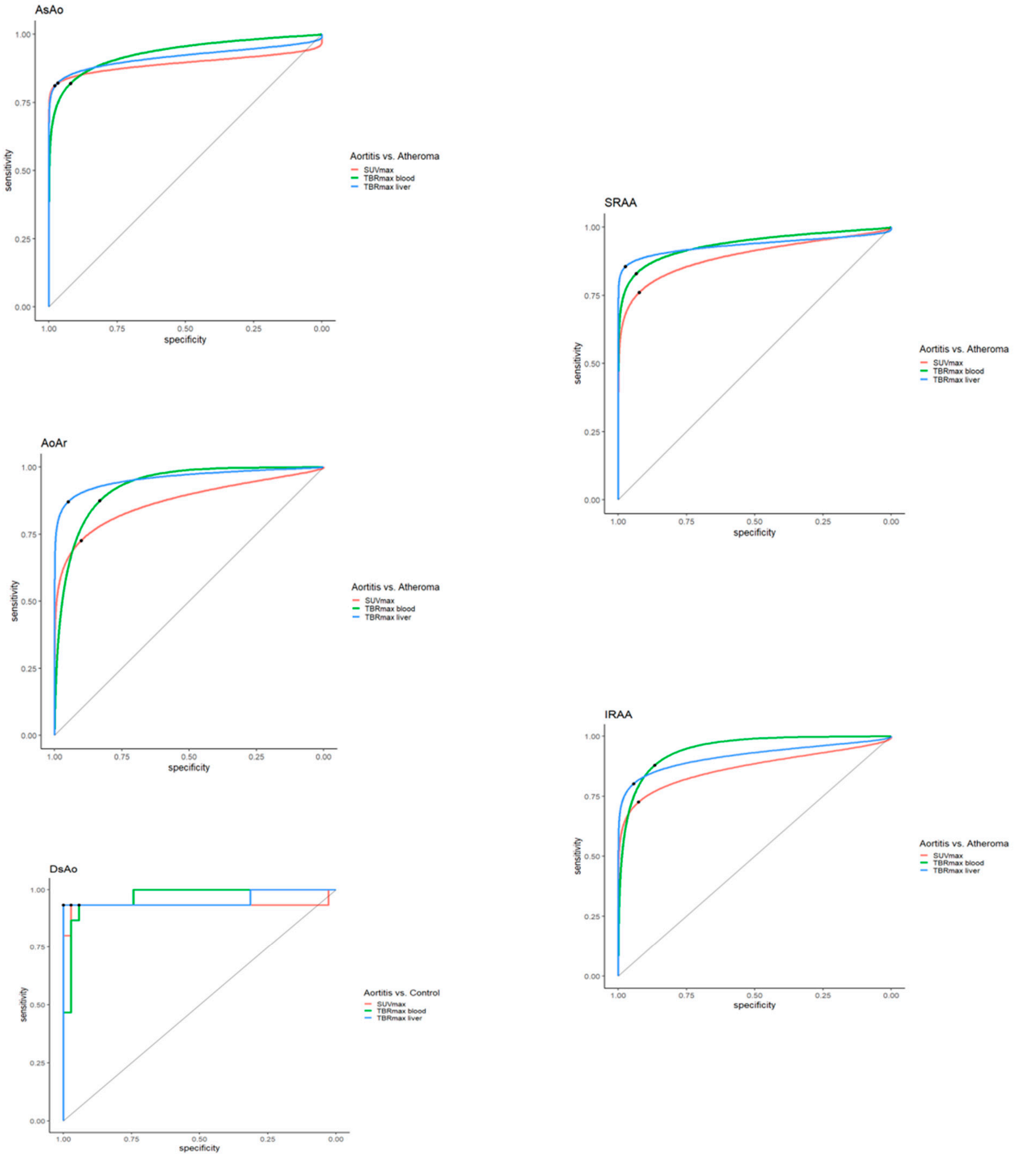

Supplemental Figure S3: ROC curve of SUV_{max}, TBR_{blood} and TBR_{liver} in the ascending thoracic aorta (AsAo), aortic arch (AoAr), descending thoracic aorta (DsAo), suprarenal abdominal aorta (SRAA) and infrarenal abdominal aorta (IRAA), according to GCA aortitis without corticosteroid and the aortic atheroma group.

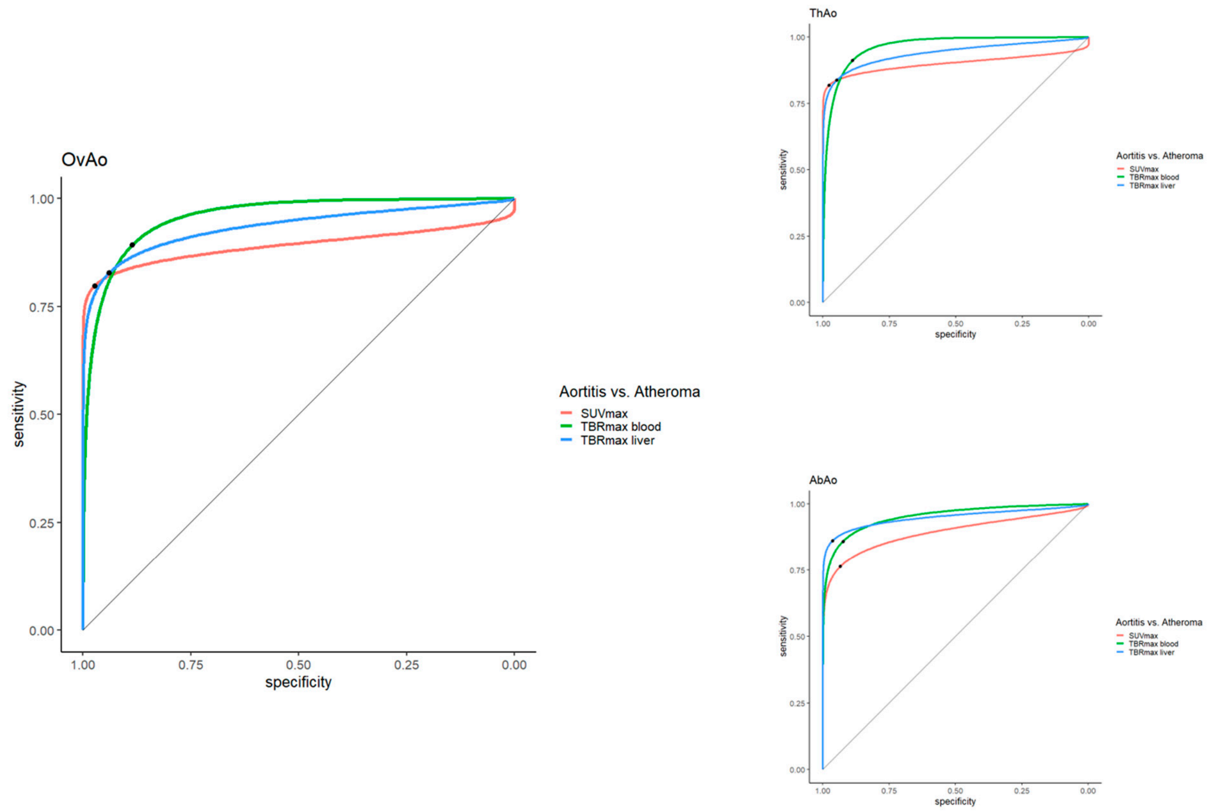

Supplemental Figure S4: ROC curve of SUV_{max}, TBR_{blood} and TBR_{liver} in the overall aorta (OvAo), thoracic aorta (ThAo) and abdominal aorta (AbAo) according to GCA aortitis without corticosteroid and the aortic atheroma group.

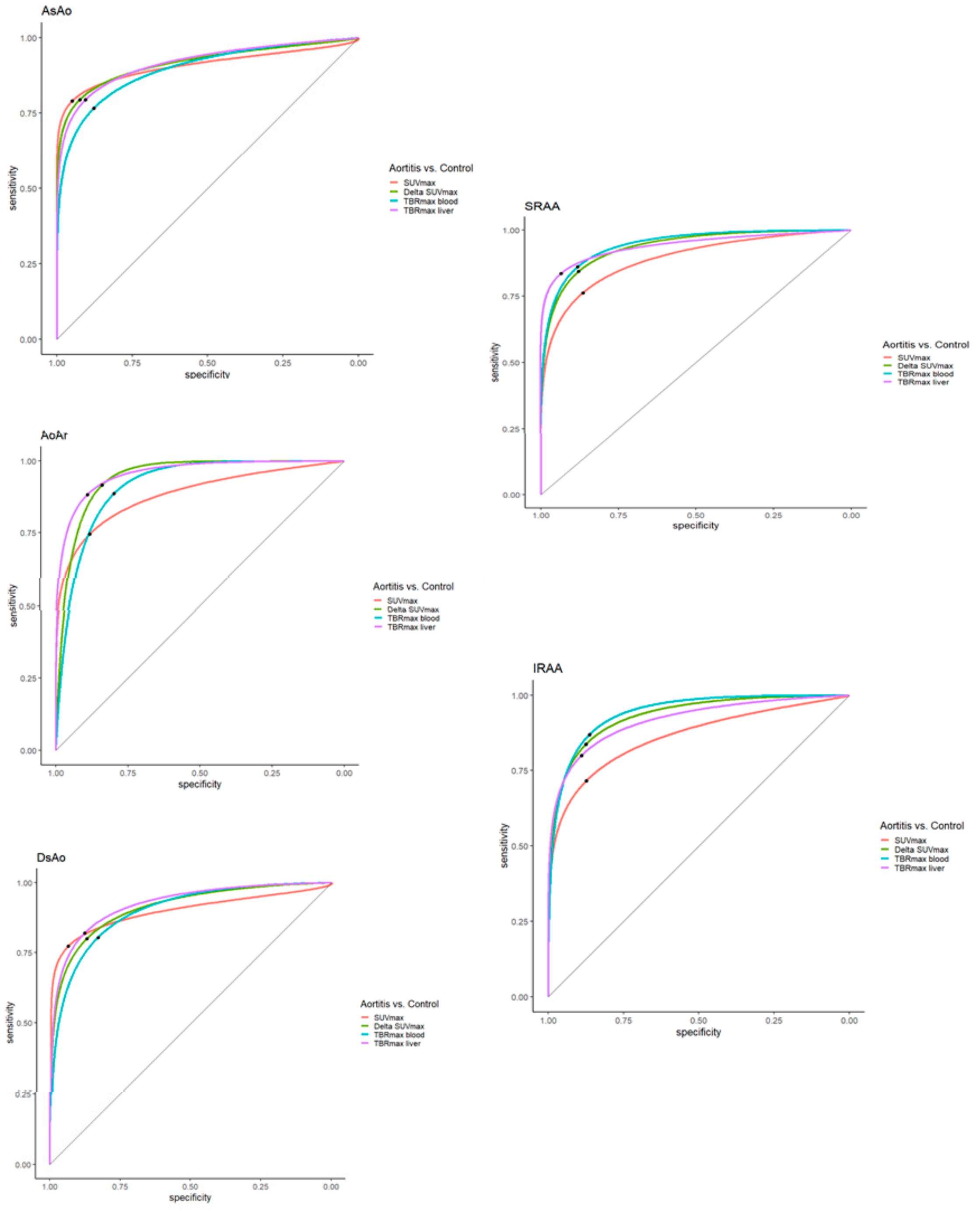

Supplemental Figure S5: ROC curve of SUV_{max}, TBR_{blood} and TBR_{liver} in the ascending thoracic aorta (AsAo), aortic arch (AoAr), descending thoracic aorta (DeAo), suprarenal abdominal aorta (SRAA) and infrarenal abdominal aorta (IRAA) according to GCA aortitis without corticosteroid and the control group.

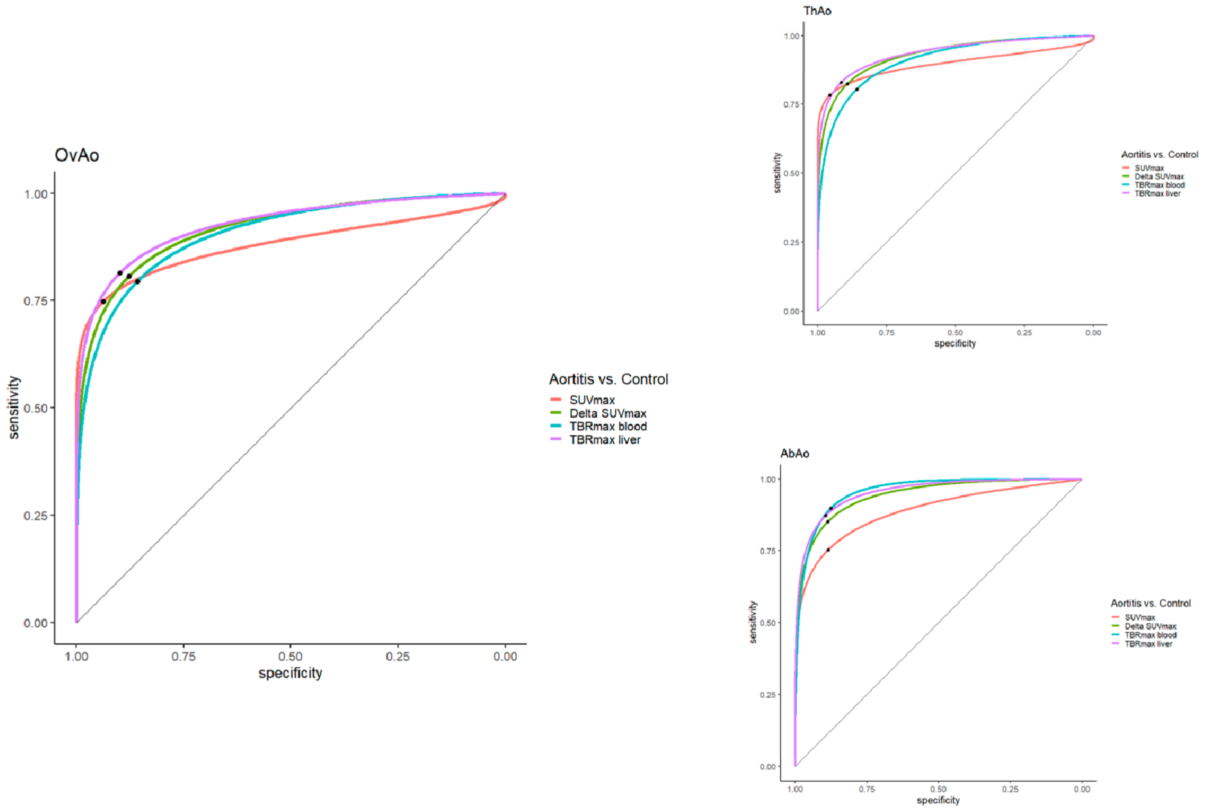

Supplemental Figure S6: ROC curve of SUV_{max}, TBR_{blood} and TBR_{liver} in the overall aorta (OvAo), thoracic aorta (ThAo) and abdominal aorta (AbAo) according to GCA aortitis without corticosteroid and the control group.

	Aortitis without GC vs. Controls			Aortitis without GC vs. aortic atheroma		
	SUV_{max}	TBR_{max} blood	TBR_{max} liver	SUV_{max}	TBR_{max} blood	TBR_{max} liver
**Ascending aorta**						
AUC	0.90	0.95	0.93	0.91	0.98	0.94
Cut-off	3.05	2.06	0.98	2.85	1.67	1.14
Specificity	0.94	1	0.97	1	1	1
Sensitivity	0.91	0.82	0.91	0.91	0.91	0.81
**Aortic arch**						
AUC	0.88	0.94	0.97	0.86	0.93	0.91

Cut-off	3.15	1.74	0.88	3.15	1.75	0.94
Specificity	0.89	0.86	0.91	0.82	0.87	0.84
Sensitivity	0.82	0.88	0.94	0.82	0.88	0.88
**Descending aorta**						
AUC	0.93	0.97	0.95	0.92	0.95	0.94
Cut-off	3.35	1.83	1.03	3.40	1.82	1.09
Specificity	0.97	0.94	1	0.91	0.87	0.97
Sensitivity	0.93	0.93	0.93	0.93	0.93	0.93
**Supra renal abdominal aorta**						
AUC	0.91	0.95	0.94	0.90	0.93	0.93
Cut-off	3.9	1.58	0.92	3.35	1.81	1.10
Specificity	1	0.89	0.94	0.93	0.97	1
Sensitivity	0.73	0.93	0.93	0.80	0.87	0.87
**Infra renal abdominal aorta**						
AUC	0.87	0.96	0.94	0.87	0.92	0.91
Cut-off	3.2	1.44	0.92	3.25	1.57	1.08
Specificity	0.97	0.86	0.97	0.94	0.86	1
Sensitivity	0.73	0.93	0.87	0.73	0.87	0.80

Supplemental Table S1: PET aortitis without corticosteroid signal vs. aortic atheroma signal receiver operating characteristic (ROC) curves analyses, and aortitis without corticosteroid signal vs. aortic signal in control ROC curves analyses in five different aortic segments (AUC: area under the curve, GC: glucocorticoids).

	Aortitis without GC vs. normal controls			Aortitis without GC vs. aortic atheroma		
	SUV_{max}	TBR blood	TBR liver	SUV_{max}	TBR blood	TBR liver
**Thoracic aorta**						
AUC	0.90	0.97	0.95	0.90	0.96	0.92
Cut-off	3.45	1.89	0.94	3.40	1.77	1.09
Specificity	0.97	0.97	0.94	0.91	0.83	0.82
Sensitivity	0.88	0.78	0.88	0.88	1	0.95
**Abdominal aorta**						
AUC	0.91	0.96	0.96	0.90	0.94	0.93
Cut-off	3.90	1.58	1.13	3.95	1.81	1.10
Specificity	1	0.89	1	0.98	0.97	1
Sensitivity	0.75	0.94	0.88	0.75	0.88	0.87
**Overall aorta**						
AUC	0.89	0.97	0.94	0.89	0.97	0.90
Cut-off	3.65	1.94	1.04	3.45	1.97	1.09
Specificity	0.97	0.94	0.97	0.90	0.93	0.96
Sensitivity	0.83	0.89	0.83	0.89	0.89	0.83

Supplemental Table S2: Aortic PET values in aortitis without glucocorticosteroid vs. aortic atheroma receiver operating characteristic (ROC) curves analyses, and aortitis without glucocorticosteroid vs. normal control ROC curves analyses in thoracic and abdominal aorta and in overall aorta (AUC: area under the curve, GC: glucocorticoids).

	Aortitis with GC vs. controls			Aortitis with GC vs. aortic atheroma		
	SUV_{max}	TBR_{max} blood	TBR_{max} liver	SUV_{max}	TBR_{max} blood	TBR_{max} liver
**Thoracic aorta**						
AUC	0.90	0.85	0.94	0.88	0.84	0.86

Cut-off	3.05	1.57	0.95	3.25	1.75	0.93
Specificity	0.83	0.66	0.94	0.86	0.81	0.77
Sensitivity	0.88	0.94	0.81	0.81	0.81	0.86
**Abdominal aorta**						
AUC	0.91	0.96	0.96	0.88	0.92	0.85
Cut-off	3.05	1.53	0.88	3.05	1.70	0.91
Specificity	0.91	1	1	0.73	0.85	0.63
Sensitivity	0.83	0.89	0.86	0.91	0.86	0.63
**Overall aorta**						
AUC	0.87	0.85	0.92	0.85	0.81	0.81
Cut-off	3.05	1.94	0.93	3.35	1.75	0.97
Specificity	0.80	0.94	0.86	0.89	0.76	0.76
Sensitivity	0.88	0.63	0.88	0.75	0.81	0.75

Supplemental Table S3: PET aortitis with corticosteroid signal vs. aortic atheroma signal receiver operating characteristic (ROC) curves analyses, and aortitis with corticosteroid signal vs. aortic signal in control ROC curves analyses in three different aortic segments, in thoracic and abdominal aorta and in overall aorta (AUC: area under the curve, GC: glucocorticoids).
